# Dulcamara in the Ovario-Uterine Sphere

**Published:** 1887-08

**Authors:** George H. Clark

**Affiliations:** Germantown, Phila.; Bureau of Materia Medica, I. H. A.


### DULCAMARA IN THE OVARIO-UTERINE SPHERE.

Geo. H. Clark, M. D., Germantown, Phila.

(Bureau of Materia Medica, I. H. A.)

Dulcamara is a remedy that has not received the attention it deserves, particularly in the affections of women.

Its application to troubles arising from damp and cold should give it prominence in these affections, and in order that what it is capable of doing may be better known, the following is offered:

In menstrual troubles it may be useful in these conditions: Menses too copious, too long, premature, retarded, scanty, too short, suppressed, pale and watery.

Into all of these the damp and cold factor enters, and where there is reason for believing that any of them have had their origin in these causes, Dulcamara should be given a prominent place in looking for a remedy.

Preceding the menses there are an erythematous eruption on the chin and nettle-rash on various parts of the body, with much itching; after scratching it burns, and is aggravated by warmth and ameliorated by cold.

During menstruation, which may be suddenly suppressed from damp and cold, there are engorgement of the breasts and eruptions on the labiæ majores.

These eruptions are of an herpetic character, and also of an eczematous nature.

Other remedies having an eruption about the pudenda, in connection with the menses, are Aurum, Causticum, Graphites, Mercurius, Petroleum, and Staphysagria.

The Aurum eruption is of large red pimples. Graphites and Mercurius also have pimples.

The Caust. and Petrol. eruptions are herpetic. Staphysagria has blotches on the labiæ majores, with itching and soreness when touched.

With the retarded menstruation of Dulcamara are the eruptions, and paleness and watery condition of the flow.

Pulsatilla has pale and watery blood, but it alternates with black blood mixed with mucus.

Kali carbonicum has soreness and redness around the parts and between the thighs, with retarded menstruation, and the blood is acrid and fetid.

Closely allied to Dulc. in scanty menses are Amm. carb., Con., Graph., Kali carb., Lach., Puls., Sep., Sulph.; and in too short menses Amm. carb., Bar. carb., Con., Graph., Lach., Puls., Sulph.

With pale and watery blood and short and scanty menses, besides Dulc., are Amm. carb., Graph., and Puls.

Suppressed menses from dampness belong particularly to Dulc. and Rhus tox.; from getting wet, Acon., Dulc., and Rhus tox.; and from getting feet wet, to Nux mosch. and Puls.

With the following indications in amenorrhœa we may expect much from Dulc.: Suppression in consequence of a cold, or in those who are affected with the various eruptions peculiar to this remedy; glandular engorgements, particularly of the mammæ; warts on the hands, and soreness of the throat. In the throat affections there are much mucus in the fauces and pressure, as if the uvula were too long, and the tonsils are apt to be inflamed. Conium has hardness of the breasts; while Phytolacca has painful and engorged mammæ, with throat symptoms prominent.

Under Kali carb., in amenorrhœa, there are frequent erysipelatous eruptions; Puls. has freckles on the face. In malignant disease of the breast, during absence of menses, Dulc. has a part; and closely allied in such affections are Ars., Bell., Carbo an., China, Cocc., Con., Graph., Kreos., Merc., Nit. ac., Sep., Sil., Staph., Thuja.

Dulc. also occupies a place in the treatment of many other affections incidental to anemia, such as bronchitis, cephalalgia, chorea, congestion of brain, diarrhœa, dysuria, epistaxis, gout, nausea, ophthalmia, paralysis, and vomiting, and these following exposure to damp and cold will be the prominent indications for Dulc.

The peculiar dysuria of Dulc. deserves notice, and is characterized by the nature of the urine, which, on standing, becomes oily, and contains a tough, jelly-like white or red mucus, mixed with blood.

The urine is milky under Cina, while with Colocynth it deposits a copious, jelly-like sediment, and there are small, hard, reddish crystals, which adhere to the vessel and are not readily removed by water.

Dulcamara also has place in the treatment of ulceration of the womb, ovaritis, vesicular vaginitis, herpetic and lichenous eruptions of pudenda, ecchymosis of breasts, and ptyriasis of, and scabs and crusts on, the nipples.

During the period of gestation it will come in for diarrhœa and for cough, accompanied by obstinate tickling in the throat and chest, which latter symptom belongs also to Conium. The Conium cough is made worse by lying down, particularly immediately on going to bed.

In suppression of milk by cold, Dulc., with Acon., Bell., Cham., Merc., Puls., and Sulph., should receive attention. And for affections arising from this cause, at this period, we have Agnus, Bell., Bry., Calc., Cina, Dulc., Lach., Lycop., Merc., Sep., Sulph., Zinc.

Where skin symptoms follow suppression of milk, Calc., Caust., Dulc., Rhus tox., and Sulph. are to be studied.

From this we can readily see that Dulc. deserves some notice, and if this paper succeeds in directing your attention to this valuable remedy, my aim will be attained.

